# Detecting momentary reward and affect with real-time passive digital sensor data

**DOI:** 10.1093/jamiaopen/ooag005

**Published:** 2026-01-23

**Authors:** Samir Akre-Bhide, Zachary D Cohen, Amelia Welborn, Tomislav D Zbozinek, Michelle G Craske, Alex Bui

**Affiliations:** Medical Informatics Home Area, University of California, Los Angeles, Los Angeles, CA 90024, United States; Department of Psychology, University of Arizona, Tucson, AZ 85721, United States; Department of Psychiatry and Biobehavioral Sciences, University of California, Los Angeles, Los Angeles, CA 90095, United States; Department of Psychiatry and Biobehavioral Sciences, University of California, Los Angeles, Los Angeles, CA 90095, United States; Department of Psychiatry and Biobehavioral Sciences, University of California, Los Angeles, Los Angeles, CA 90095, United States; Department of Psychology, University of California, Los Angeles, Los Angeles, CA 90095, United States; Department of Radiological Sciences, University of California, Los Angeles, Los Angeles, CA 90024, United States

**Keywords:** digital phenotyping, wearable electronic devices, machine learning, ecological momentary assessment (EMA), depression

## Abstract

**Objectives:**

This study explores the capability of passive digital sensor data from smartphones and smartwatches to predict self-reported ecological momentary assessments (EMA) of affect, motivation, interest, and pleasure in activities in an unseen test sample.

**Materials and Methods:**

Data were collected from 245 depressed participants with high-to-low anhedonia (195 train, 50 test) generating 23 812 EMA sessions. Machine learning models were used to assess the ability of behavioral and physiological features, aggregated over windows of 15 minutes to 3 hours, to predict momentary subjective states.

**Results:**

For 12 of 15 EMA questions asked, machine learning models exceeded random chance in the fully-held-out test sample, suggesting detectable signals between passive measures and subjective states. Dependent on the sensor type, the optimal aggregation periods ranged from 15 minutes to 3 hours, with generally at least two hours of data being required. Subgroup analyses revealed variations in model performance by demographics, depression severity, and anhedonia severity.

**Conclusion:**

This study establishes the feasibility of using passive digital sensing to detect momentary subjective states, providing a baseline for scalable, non-invasive mental health monitoring

## Introduction

Passive digital sensor data can capture real-time behavioral and physiological data, which is increasingly valuable in continuous mental health monitoring.^1^ These data from consumer smartphones and wearable devices are frequently compared to classic (ie, retrospective) self-report surveys in the context of monitoring mental health.^2^ However, such passive digital sensing provides the ability to objectively examine much more granular time series data than does the broader, retrospective scope of weeks and months typically covered by self-reports. Ecological momentary assessment (EMA) methods, also known as experience sampling, enable the collection of contextually sensitive, moment-to-moment self-reported mental state data from participants at a much finer temporal resolution.^3^^,^^4^ The use of EMA as an outcome measure to assess digital sensing aligns more closely with the inherent time scale of these technologies.

Prior studies have found that 3 or fewer hours of passive sensing data—combining heart rate, HRV, skin conductance, and motion measurements during EMA responses—are associated with momentary stress,^5^^,^^6^ paranoia,^7^^,^^8^ social interactions,^9^ mood,^6^^,^^10–13^ tiredness,^14^ and inhibitory control.^15^ These studies leverage a form of within-participant (idiographic) or mixed modeling approaches where models are trained with some data from a given participant prior to evaluation. Studies such as Taylor et al have found that for tasks such as mood prediction with passive sensing data accuracy can decrease from 78.7% to 59.4% when going from mixed modeling approaches to nomothetic.^16^ Similarly, Aalbers et al found that idiographic approaches increased performance by up to 50.45% relative to nomothetic baselines in predicting stress.^17^

However, it remains unclear which signals from passive digital sensor data can reliably indicate subjective states across different individuals, rather than only providing insights specific to each individual in a personalized context.^18^ As such, our work centers on nomothetic modeling where models are evaluated on data from participants they have not seen before, with no input features other than those collected from passive digital sensors. The goal of this harder task is to identify if any more generalizable signal may exist between digital sensing measures and momentary subjective state without personalization. Importantly, if successful, this work would allow for the application of passive sensing models to detect momentary affect in new individuals without the need for any self-report or model tuning.

This study examines whether digital sensing data without prior participant-specific information can detect self-reported momentary affect and reward processes—including motivation, interest, and pleasure in activities—as captured in EMAs. We use passive data from smartphones and smartwatches alongside EMAs to assess whether individuals’ behaviors and physiological patterns can detect subjective experience. This approach provides an understanding of the behaviors and physiological factors associated with specific changes in affect and reward functioning, which, when chronic and extreme, become disordered. Our analysis utilizes data from 245 participants (split into a training sample of *N* = 195 and a held-out test sample of *N* = 50) who collectively generated 23 812 EMA sessions. The sample was drawn from the Operationalizing Digital PhenoTyping in the Measurement of Anhedonia (OPTIMA) study, which recruited individuals who were moderately-to-severely depressed with high-to-low anhedonia severity.

There are two key considerations when creating features from passive digital sensor data. First, to better understand the time course of the association between different passive features (eg, physiological processes like heart rate and behavioral features like “steps taken”) and momentary report of affect and reward, we investigate performance differences based on an aggregation period for sensor data prior to an EMA response, ranging from 15 minutes to 3 hours. Second, we assess whether incorporating passive digital sensor data from the previous night’s sleep enhances model performance. We hypothesize that passive digital sensor data can reliably predict (We use the term ‘predict’ to describe the process of identifying one measure (subjective momentary state) based on another set of features (passive digital sensing). This usage aligns more closely with detection rather than prediction in the traditional sense of temporal forecasting.) subjective momentary states and aim to determine the optimal feature creation methods, including aggregation period and data type, that maximize model performance in predicting these states.

## Methods

The data for this analysis were obtained from 342 participants who were monitored via passive digital sensing and self-reported measures over 13 weeks as part of the Operationalizing Digital PhenoTyping in the Measurement of Anhedonia (OPTIMA) study investigating features of anhedonic depression, which collected data between October 2022 and April 2024. Self-reported depression severity is taken from a subset of the responses to the Patient Health Questionnaire-14 (PHQ-14) (see Depression Symptom Response Project OSF site: https://osf.io/j6r3q/) with the total score intended to replicate the PHQ-8, and self-reported anhedonia severity is assessed via the Positive Valence Systems Scale (PVSS).^19^

The OPTIMA study is part of the Wellcome Leap Multi-Channel Psych Program, a consortium of studies focused on anhedonic depression. A subset of 245 participants was used in this analysis. This subset was selected based on: having greater than 30% of expected days with EMAs containing at least 2 of 5 responses, more than 30% of the first 90 days in the study with at least one log of heart rate, and greater than 0 variability in the individual’s EMA responses (at least one EMA item has greater than 0 variability per participant). This 30% threshold was chosen to ensure that each participant had sufficient variance in both sensor data and EMA responses for model training and evaluation, while excluding participants with device errors or low study protocol compliance.

The participants were screened to all be right-handed, but no instructions were given as to whether watches would be worn on the nondominant hand (or not). The participants were given the Apple watch series 7 or higher and used their own iPhone. This analysis is exploratory and was not preregistered, but its reporting incorporates relevant expected information described in the “Template for studies using passive smartphone measures” by Langener et al^20^

All research study activities were reviewed and approved by the UCLA Neuroscience institutional review board MIRB3 (IRB00004473). All research study participants signed informed consent for the study protocol (#22-000059) prior to participation.

### Ecological momentary assessment processing

EMAs were administered to participants via the UCLA Depression Grand Challenge Study App (DGC Study App) for three separate 8-day bursts (5 times per day) during the study (at baseline, week 6, and week 12). An EMA session is an event associated with administering the full set of EMA questions. The 15 EMA items used in this analysis correspond to either affect or reward functioning. The EMA session begins by instructing participants: “The following questions will ask you to describe your feelings and experiences right now. “Right now” means right before you began this survey.

Affect-related EMA items are of the form “How ___ do you feel right now?” where the blank is one of nine items:

1. Sad
2. Stressed
3. Anxious
4. Annoyed/Irritated
5. Energetic
6. Happy
7. Motivated
8. Engaged
9. Lonely

The participants responded on a 5-point Likert scale with the following mapping:

1. Not at all
2. Slightly
3. Moderately
4. Very
5. Extremely

All affect-related items are converted to binary responses if their answer is greater than 1 (not at all). This results in affect-related EMA items being converted to the classification of no endorsement vs any endorsement. Reward-related items are binary (true or false) responses. The questions are in the format, “Right now, I…” and end with (underlines represent the term used in figures and tables to describe the EMA item):

1. am looking forward to an upcoming activity (anticipatory)
2. am feeling good after doing something (consummatory)
3. am putting effort into planning something
4. could be doing something positive but am not because I don’t think I’d enjoy it (inactive enjoy)
5. could be doing something positive but am not because it feels like too much effort (inactive effort)
6. am feeling a sense of meaning and purpose.

The presentation order was randomized within the affect and reward item blocks. An The participants were instructed that they had 30 minutes from the time the EMA was sent to submit their responses. EMA sessions that lasted shorter than 10 seconds or longer than 5 minutes were not used in the analysis. This filtering results in the removal of 461 (1.9%) sessions, with 23 812 remaining. After quality control, the total number of questions answered is 356 255 (between 23 687 and 23 793 responses per EMA question investigated). A comparison of OPTIMA participant EMA responses to a more clinically and demographically representative U.S. population is described in the supplemental materials and shown in Figure S1 and Table S1.

### Dataset split

To ensure an even distribution of important participant-level characteristics between the training data sample and the test set, the participants were split to ensure approximately equal distributions across 10 variables, with an approximately 80-20 split of participants (195 train, 50 test). This split leads to 19 016 EMA sessions in the training data and 4796 in the testing data. The full list of variables used to split the data into training and test sets were:

1. Age: Distribution of integer years
2. Sex at birth: Male or female
3. Race and ethnicity: Operationalized as identifying as non-Hispanic white or not
4. Study participation: Number of study timepoints with self-reported data available
5. PHQ-14 total score (which aligns with the PHQ-8 total score) at the end of the study
6. PVSS total score at the end of the study
7. Availability of EMA responses: Percentage of expected days (24) within the first 90 days of the study with at least two EMA responses
8. Availability of watch data: Percentage of days with at least one heart rate entry within the first 90 days of the study
9. Availability of sleep annotation data: Percentage of days with sleep duration calculatable within the first 90 days of the study
10. Variance of EMA responses: Average standard deviation of EMA responses across all 15 items used as prediction targets

Assignment to training or test splits was performed via the automated randomization of multiple traits for study design (ARTS) package.^21^ The distributions of the variables between the training and test splits are shown in Figure S2.

### Passive sensor features

Passive sensor features are created relative to each timestamp when each EMA session starts. A total of 29 momentary features from vital signs, activity, and ambient noise are generated during the time window prior to the start of the EMA session, and an additional 11 features related to sleep quality from the previous night are generated. To investigate whether the prior night’s sleep augments predictive performance, we compare models using only the 29 momentary features against models that include both momentary and sleep quality features. Details of heart rate monitoring from consumer devices are reported where possible in accordance with guidelines from Nelson et al^22^ These features are as follows:

1. Vital signs (13 features): Prior to aggregation, vital signs are resampled as the median at 5-minute intervals to account for dynamic sampling. Heart rate is the most frequently sampled vital sign (the median sampling time is every 2 minutes), so more aggregations are run for it than others. The mean, standard deviation, minimum, maximum, number of entries, slope, and intercept (from linear model fit) are used as features. For respiratory rate, heart rate variability (measured as the standard deviation of the N-to-N interval), and oxygen saturation, mean and number of entries are captured.
2. Activity (12 features): Active energy expenditure, basal energy expenditure, exercise time, and step count, are aggregated by taking the sum value, count of entries, and sum duration of entries.
3. Ambient noise (4 features): Audio exposure events from Apple HealthKit are aggregated by taking the total number of entries, the number of 5-minute intervals with entries, the mean decibel level, and the sum duration of entries. Ambient noise is sampled at a median rate of once every 30 minutes.
4. Sleep (11 features): Sleep features are calculated on the basis of HealthKit sleep annotations from 3pm the prior day until 3pm of the day of EMA assessment. We generate sleep duration, bedrest duration, sleep efficiency, sleep onset latency, sleep onset, sleep offset, bedrest onset, bedrest offset, number of nighttime awakenings, duration of awakenings, and average noise during bedrest.

The median time difference between samples for vitals, activity, and ambient noise is shown in Figure S3 to provide context for what data may be missing on shorter time aggregation windows.

Entries where calculation of mean heart rate was not possible (no heart rate entry was found) were excluded from the analysis. Reporting of a heart rate measurement from the watch is used as a proxy for watch wear. Other missing features are imputed as the median value per feature in the training set. The percentage of missing features per aggregation period is shown in Figure S4 after the removal of EMA sessions, as described in the Ecological Momentary Assessment Processing section.

### Machine learning modeling

To predict the EMA binary responses using passive sensor-derived features, we assessed multiple machine learning models. Specifically, we aimed to determine whether these features could accurately predict participants’ responses to EMA items. The models evaluated included gradient boosting classifier (XGBoost), random forest (RF), light gradient boosting classifier (LGBM), logistic regression with L1 regularization (LRL1, also known as LASSO), and logistic regression with L2 regularization (LRL2, also known as Ridge).

The data preprocessing pipeline included scaling features using a standard scaler and imputing missing values via simple median imputation before model prediction. All models were implemented using scikit-learn version 1.2.2.^23^ Hyperparameter tuning and model selection were performed using Automated Machine Learning (AutoML) via the fast and lightweight AutoML library (FLAML) version 2.1.2^24^ optimizing for AUROC value. The AutoML process was applied exclusively to the training data on the full ML pipeline (including scaling and imputation), employing 5-fold stratified grouped cross-validation. Grouping was implemented to ensure that participants in the training folds were not present in the validation folds during hyperparameter tuning, thus preventing data leakage. The time budget for AutoML was set to 200 seconds per model, and early stopping was utilized to optimize the tuning process. To further prevent data leakage and ensure the robustness of our evaluation, the final performance of the selected models was assessed on a fully held-out test set comprising 50 participants who were not included in any part of the model training or hyperparameter tuning process.

### Model evaluation

The best performing model per EMA response from the training phase were evaluated for performance on the test dataset of 50 participants and 4796 EMA responses via the metric of area under the receiver operating characteristic curve (AUROC). To account for the unequal number of EMA responses per participant and to generate confidence intervals, a bootstrapped sampling approach was taken using 1000 bootstrapped samples. Each bootstrapped sample consisted of 100 EMA responses per individual in the test set sampled randomly with replacement, and for each bootstrapped sample, the AUROC was calculated. Bonferroni adjustment is used when calculating confidence intervals with a family wise error rate (FWER) set to 0.05 across 150 comparisons (15 outcomes, 5 aggregation window sizes, 2 feature sets). The Bonferroni adjusted alpha value ($\alpha_{bf}$) was set to $\frac{0.05}{150}$ and confidence intervals determined as the percentile of bootstrapped AUROC values between $\frac{\alpha_{bf}}{2}$ to $1-\frac{\alpha_{bf}}{2}$. For the best performing models per outcome that had original Likert scale responses, an additional evaluation was done to investigate performance on a subset of the test set where responses were 1 or 5 (extreme values only).

The best performing model per outcome with respect to aggregation duration (15 minutes, 30 minutes, 1 hour, 2 hours, or 3 hours) and feature set (momentary features, or momentary and sleep features) was selected per detected outcome. For these models, performance was evaluated stratified by the following user characteristics: age, race and ethnicity, sex at birth, anhedonia, and depression. Each characteristic was converted to a binary variable. Age, anhedonia severity, and depression severity were split based on whether the participants were above or below the median value in the test set. Anhedonia was assessed as participants with end-of-study PVSS scores less than the median (5.95), and depression was assessed as end-of-study PHQ-14 scores greater than the median (13.5). Racial-ethnic background is converted into a binary by determining whether an individual self-identifies as non-Hispanic white. A comparison of the AUROC between each group was performed via the Wilcoxon signed-rank test. The feature importance for the best performing model is assessed via SHapley Additive exPlanation (SHAP) scores.^25^

## Results

### Model performance

**Figure 1.**
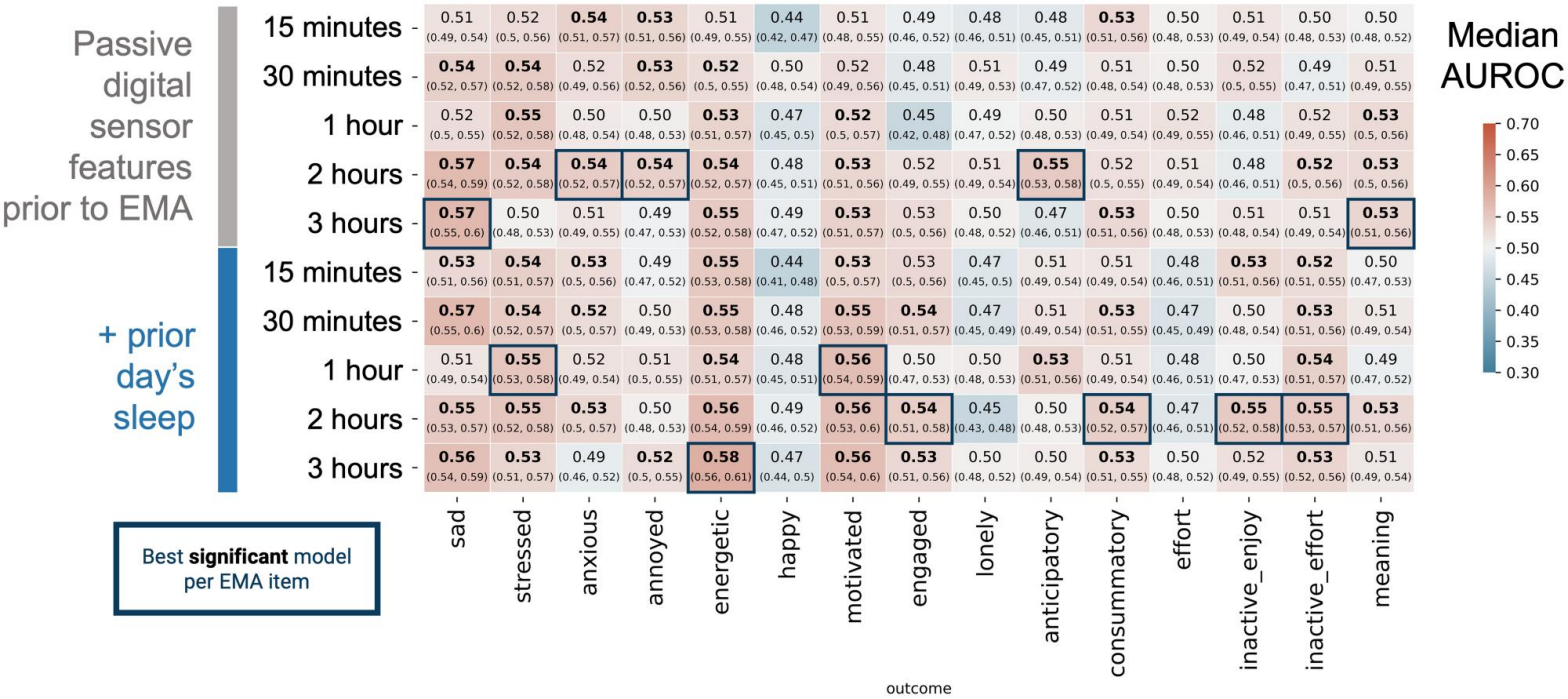
Model performance using passive digital sensor data to detect EMA item response (median AUROC ± 95% Bonferroni-adjusted bootstrapped confidence interval) on the held-out test set of 50 users for each feature set (rows) and each outcome (columns). Y-axis separates different feature sets used as inputs to the models based on the aggregation window (15 minutes to 3 hours) and the inclusion of passively sensed sleep from the night before. Bold values indicate model AUROC performance for an outcome where *P* < .05 for testing that AUROC > 0.5 after Bonferroni adjustment of Mann Whitney *U*-test. Blue outline indicates highest median AUROC for significantly performing model for a given outcome column in the test set. Note that apparent equal values only appear as such due to rounding to two decimal places. AUROC = Area under the receiver operator characteristic curve.

Our findings indicate that machine learning models can predict EMA responses at levels significantly above random chance (AUROC > 0.5, confirmed by Mann–Whitney *U* test with Bonferroni correction at FWER = 0.05) for 12 out of 15 EMA outcomes. Models using passive data could detect momentary self-report of sadness, stress, anxiety, annoyance, energy, motivation, engagement, anticipatory and consummatory reward, inactive enjoyment, inactive effort, and eudaimonic meaning, but not happy, lonely, or effort. In this context, ‘inactive enjoyment’ refers to the response to the question, “Right now, I could be doing something positive but am not because I don’t think I’d enjoy it,” while ‘inactive effort’ reflects the response to a similar question ending with “…because it feels like too much effort.” The 5-point Likert scale response to EMA mood items (“How X do you feel right now?”) were binarized (“Not at all” vs “Slightly” to “Extremely”). See Methods section for additional details on EMA question content. Table 1 and Figure 1 show that the AUROC reliably exceeded 0.5 for 12 EMA outcomes, demonstrating that machine learning models can detect specific subjective states beyond random chance. Additionally, for the Likert scale response items we see that the “sad” EMA has a performance of AUROC 0.710 when limiting the test set to only responses of value 1 or 5 (Table S4). Model training set performance is available in Figure S5 and Table S3.

**Table 1.** Best performing model at detecting each EMA item using passive digital sensing data. Performance reported as median AUROC value across 1000 bootstraps in the test set. Train and Test N refer to number of available EMA responses with associated passive digital sensor data in the training and testing sets. AUROC = Area under the receiver operator characteristic curve.

EMA item	Feature set	Model	AUROC (95% CI)	Train *N*	Test *N*
Sad	Momentary 3h	XGBoost	0.573 (0.547, 0.602)	17 707	4412
Stressed	With Sleep 1h	LightGBM	0.554 (0.526, 0.582)	17 712	4413
Anxious	Momentary 2h	Random Forest	0.545 (0.518, 0.572)	17 719	4412
Annoyed	Momentary 2h	LightGBM	0.542 (0.520, 0.567)	17 715	4412
Energetic	With Sleep 3h	Random Forest	0.586 (0.559, 0.609)	17 713	4411
Happy	Momentary 30Min	LightGBM	0.508 (0.481, 0.537)	17 719	4410
Motivated	With Sleep 3h	Random Forest	0.567 (0.538, 0.599)	17 718	4413
Engaged	With Sleep 2h	XGBoost	0.544 (0.508, 0.578)	17 717	4413
Lonely	Momentary 2h	LightGBM	0.516 (0.489, 0.541)	17 719	4412
Anticipatory	Momentary 2h	Random Forest	0.551 (0.526, 0.576)	17 647	4402
Consummatory	With Sleep 2h	Random Forest	0.543 (0.515, 0.573)	17 640	4399
Effort	Momentary 1h	Random Forest	0.520 (0.493, 0.546)	17 641	4401
Inactive_enjoy	With Sleep 2h	XGBoost	0.552 (0.523, 0.580)	17 640	4405
Inactive_effort	With Sleep 2h	Random Forest	0.551 (0.526, 0.571)	17 633	4401
Meaning	Momentary 3h	XGBoost	0.537 (0.510, 0.562)	17 640	4403

**Figure 2.**
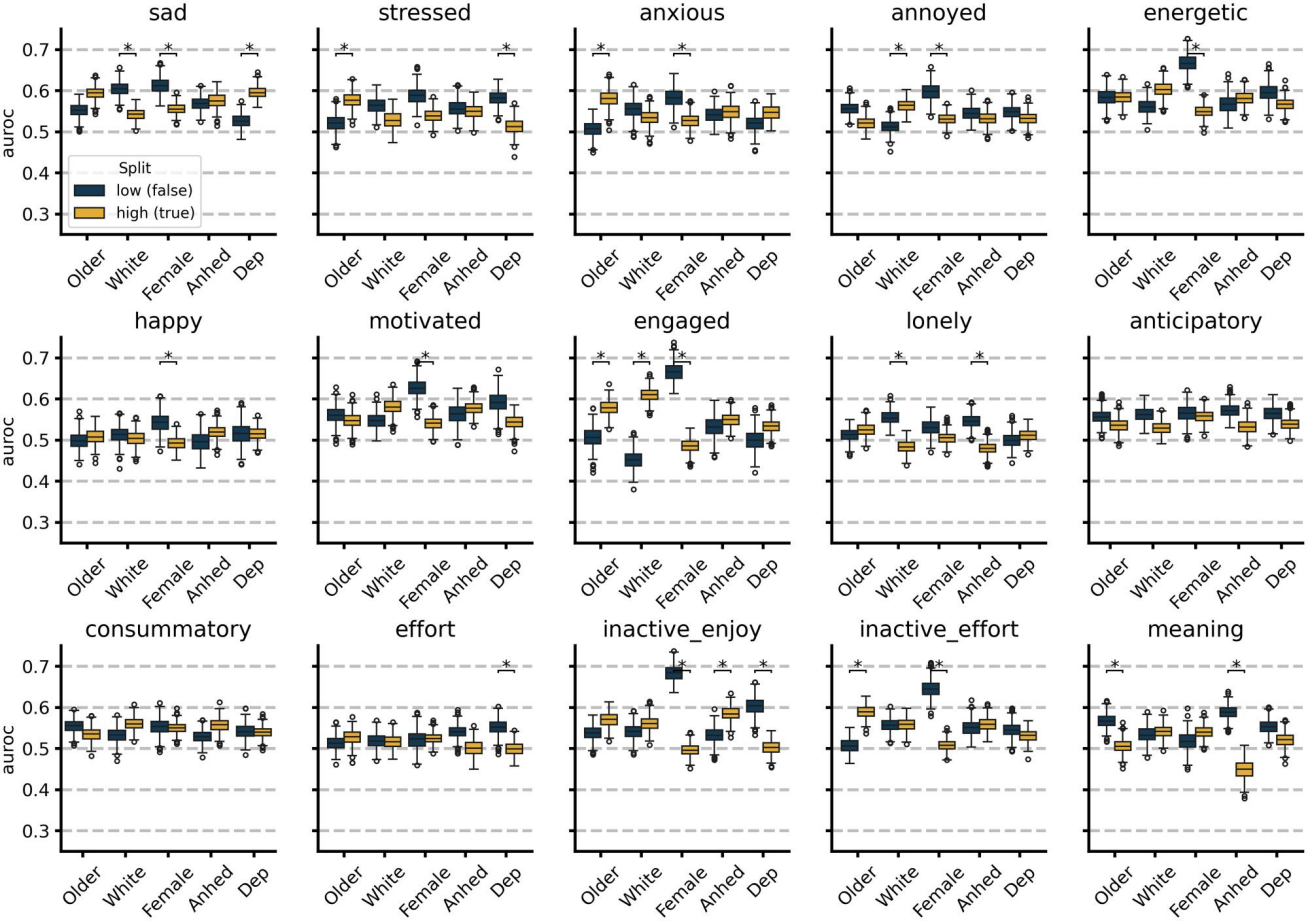
Performance of EMA detection models split by clinical and demographic parameters. * Indicates median difference between low (false) and high (true) group are >0.05 AUROC and Wilcoxon rank sum test Bonferroni adjusted *P* < .05. Age (axis label “Older”) is split at 29.5 years old; racial ethnic group (axis label “White”) is split based identification as non-Hispanic White (true group) or all other racial-ethnic groups (false group); sex-at-birth (axis label “Female”) is split by those who self-report a sex-at-birth of female (True group) or male (false group). Anhedonia (axis label “Anhed”) high/true group is determined by a PVSS total score < 5.95 and depression (axis label “Dep”) is determined by a PHQ-14 total score ≥ 13.

For the best performing model per EMA outcome, performance is assessed stratified by age, race and ethnicity, sex at birth, depression severity, and anhedonia severity. Model performance split by these characteristics is shown in Figure 2. Performance is notably higher in males for 7 of 9 affect-related EMA items (sad, anxious, annoyed, energetic, happy, motivated, and engaged) and 2 of 6 reward-related EMA items (inactive enjoy and inactive effort). Stressed, anxious, engaged, inactive effort, and meaning EMA items are better detected in older participants. The highest performing item is inactive enjoy for males with an AUROC of 0.684. Notably for the meaning EMA item, not only is performance better in those with lower anhedonia severity, performance appears inverted (AUROC< 0.5) for those with higher anhedonia.

**Figure 3.**
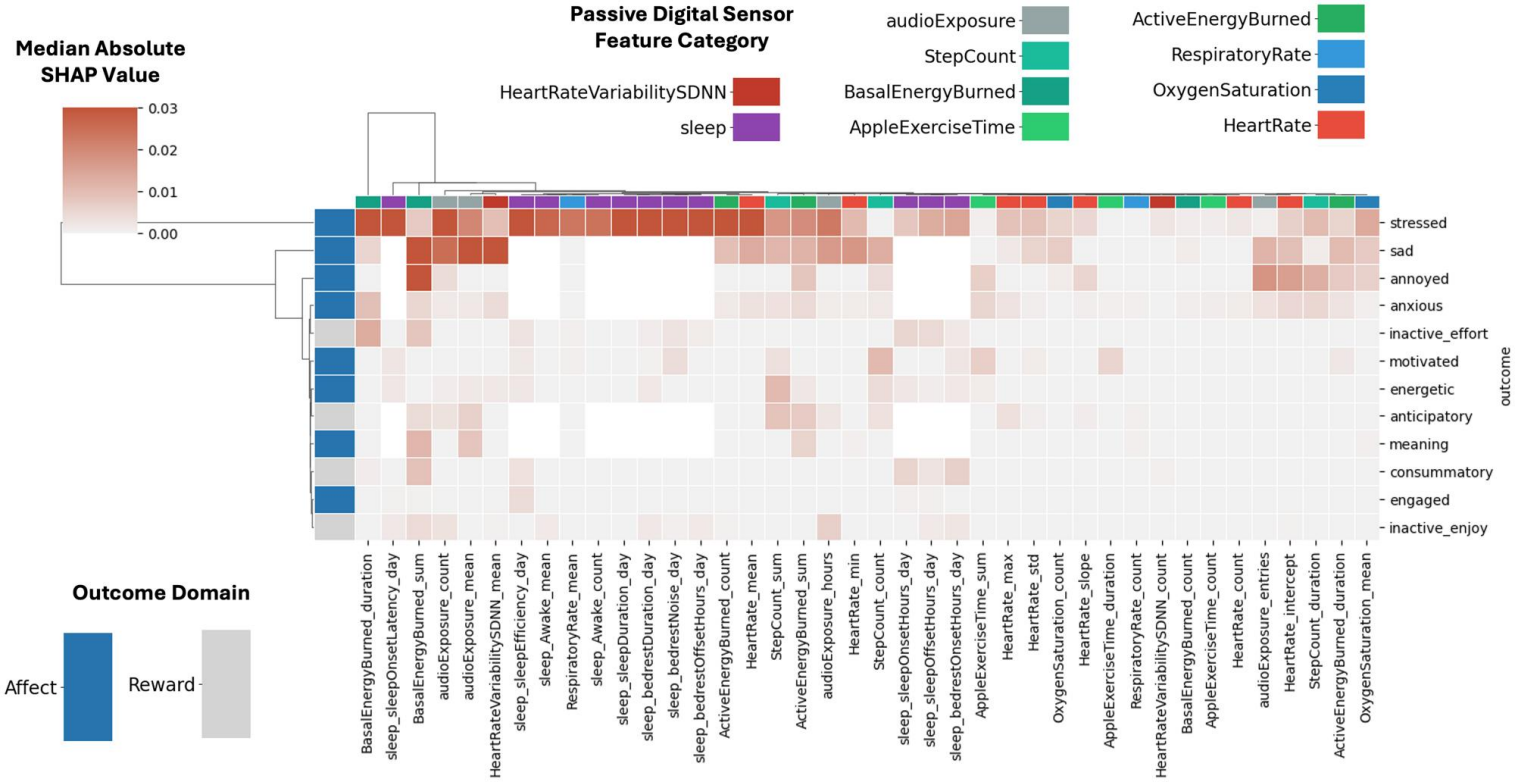
Clustered heatmap of the median magnitude of SHAP feature importance for models using passive digital sensor features (*x*-axis) to detect EMA outcomes (*y*-axis). Higher values indicate more importance of a given feature to a model. Row colors indicate if item is related to affect or reward functioning. Column colors indicate type of sensor feature. Feature importance scaled to maximum per outcome to enable easier visual comparison. Range of colomap capped from to 0.03.

**Figure 4.**
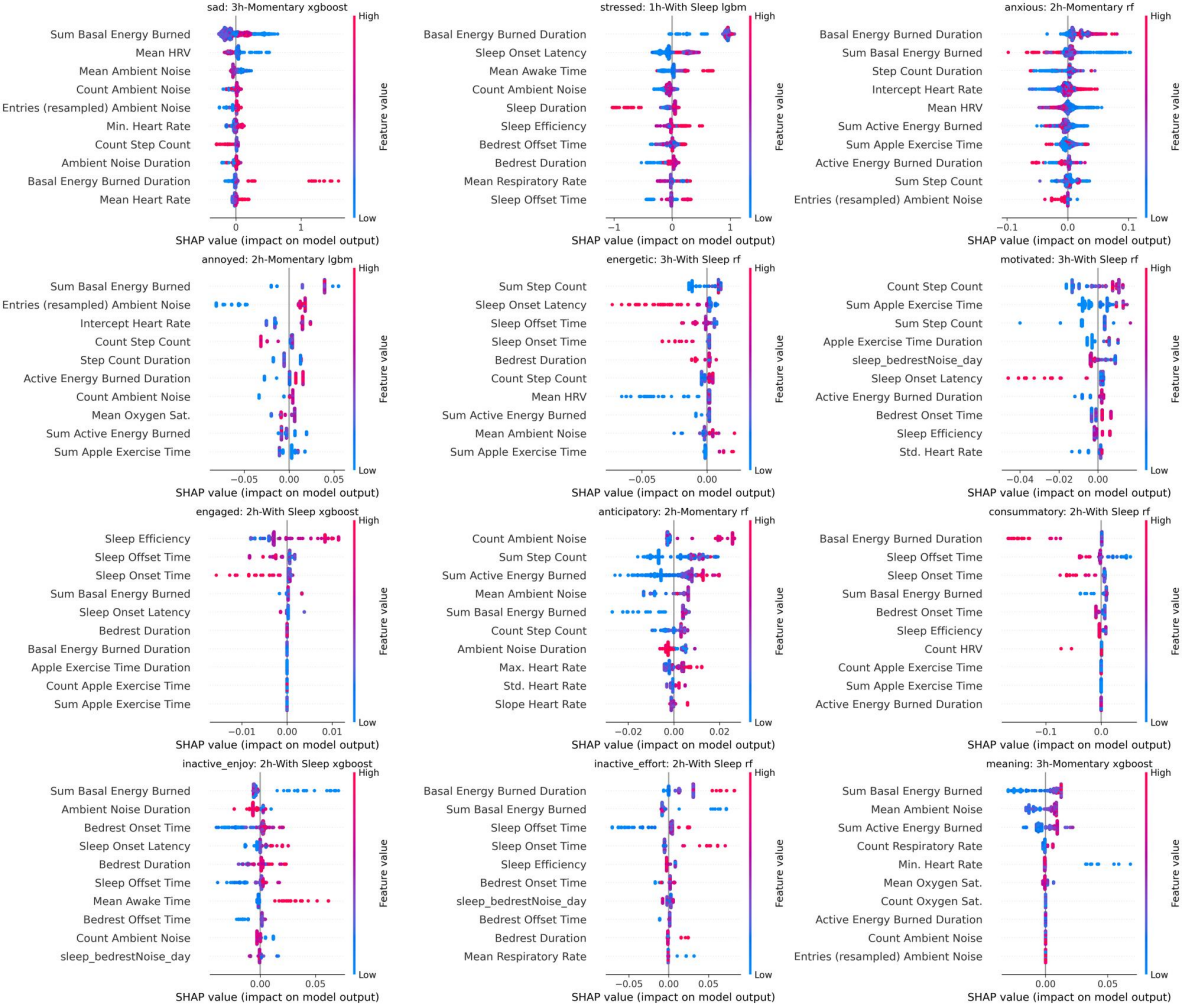
Feature importance via SHAP value for models EMA item endorsement. Each dot represents a prediction within the held out test set. Color indicates relative feature value (eg, higher mean heart rate is red, lower mean heart rates is blue). SHAP value indicates how influential a feature was to a single prediction. The sign (positive or negative) of the SHAP value denote whether importance was for classifying as negative or positive class (low vs. high class). Magnitude of the SHAP value indicates its importance to the model for a prediction.

To determine which features are consistently important across models predicting different EMA outcomes, SHapley Additive exPlanation (SHAP) analysis was performed on the 12 outcomes whose model performance was significantly greater than an AUROC of 0.5 (Figure 3). Notably, basal energy expenditure, environmental audio, and heart rate variability features consistently rank near the top features for detecting most EMA responses. A more detailed view per model of SHAP values can be seen in Figure 4.

## Discussion

Our findings suggest that passive digital sensor data can predict subjective momentary states, with model performance surpassing random chance for 12 out of 15 EMA items. No single time window consistently outperformed others across all affect and reward items, aligning with previous findings on depression self-report prediction.^26^^,^^27^ This finding suggests that the optimal aggregation period depends on the specific EMA outcome and combination of sensors being leveraged, requiring more nuanced analyses when designing such studies.

For most models, an aggregation period of at least two hours was necessary for optimal results, though this duration could decrease with higher sampling frequencies of passive sensor data, such as for HRV. Missing sensor data are prevalent, especially during smaller aggregation windows for vital signs such as respiratory rate and heart rate variability. This issue is a limitation of the sampling frequency from the consumer wearable device, which occurs dynamically. Future work could benefit from investigating how feature specific aggregation windows may improve predictive performance. Details on sampling frequency and sensitivity of models to missing data are reported in the supplementary materials in Table S2 and Figure S3. Another pattern that emerged (see Table 1) was that none of the best performing models were linear, suggesting that the relationship between subjective momentary state and passive sensor data is better characterized by accounting for non-linearities and higher-order interactions.

Including sleep data from the prior night enhanced performance for items like ‘energetic,’ where four of the top five features were sleep-related (see Figure 4). Consistent with expectations, elevated heart rate was predictive of the ‘anxious’ EMA item. Unexpectedly, basal energy burned emerged as a prominent feature in most models. Typically, basal energy burned is an estimate of the energy expended while at rest, calculated as a static variable with the Harris-Benedict equation using age, sex-at-birth, body weight, and height.^28^ HealthKit, however, reports a dynamic version using a proprietary method developed by Apple, which appears to vary throughout the day. Also relevant is the possibility that these models (trained only using passive sensor data) could be learning ‘sex at birth’ from the basal energy burned feature as the baseline value is significantly different across males and females in Figure S6.

The significant differences in model performance based on symptom severity and demographic characteristics have implications for future modeling efforts. Many models perform better for older and male participants. This is not the result of overrepresentation of older or male participants in the dataset, as only 32.7% of the participants used in the analysis (both training and test) are male and our definition of “older” was based on the median age in the test set of 29.5 years old. There is additionally no significant difference in the item response distribution between males and females in the test set for items that have significantly different performance except for the inactive effort EMA item. The performance discrepancies suggest that (for these models) the measurable behaviors from digital health devices, as assessed in this study, are most informative for the older male population, potentially reflecting a need for expanded sensors and features to enable broader detection of these items. The items for which performance is better in the low depression severity group (stressed and effort) may represent models that can better generalize to a more general (less depressed) population, as shown in Figure 2.

Markedly, recent work from Adler et al confirms that the relationship between smartphone sensed behavior and depression risk can even invert in different demographic groups.^29^ Similarly, we observe not only variations in model performance across different participant groups but also an inverse relationship in several portions of Figure 2. We specifically find this inversion for more anhedonic participants when detecting ‘meaning’ and non-White Hispanic participants when detecting ‘engaged’. These observations serve as a baseline for building future models that can leverage participant-specific information to further enhance model performance. Future work should also explore novel approaches that transcend the idiographic-nomothetic divide.^30^

Models in passive digital sensing for mental health are often trained in a within-person fashion (eg, idiographic, or, n-of-1) because performance without personalization can be low due to sample size restrictions and the complexity of the detection task.^31–33^ The sample size available in the OPTIMA study enables the use of nomothetic models to begin understanding what signals from passive digital sensing data may generalize across individuals. Nomothetic models have the benefit of being applicable with no prior labeled training data on a new participant allowing for use on data sets that only have passive digital sensor data and no EMA available. This can be useful in population health settings to gain novel insight into the subjective state of groups of individuals. Critically, for the models developed in this study, performance is low, and it will be important to consider the population the model was trained on before applying to entirely new datasets. As such, the findings of this work serve as a baseline demonstrating that passive digital sensing data is generating an independently useful signal in understanding subjective state, which personalization can then build upon. Furthermore, by exploring performance differences by demographic characteristics, depression severity, and anhedonia, we find that these parameters significantly affect model performance and begin to explain some of the characteristics that drive the heterogeneity associated with linking behavior to mental health outcomes.

We found that it is possible to detect responses to subjective momentary assessments of affect and reward functioning using only passive data and, importantly, without any prior information on a participant. As this approach precludes the need for ongoing collection of burdensome person-specific training data, our findings represent an early step toward enabling scalable continuous passive mental health monitoring and just-in-time-adaptive-interventions for the large existing user base of consumer wearable devices and smartphones.^34^ Another goal of this work was to determine the optimal time window across which to aggregate passive sensor data relative to EMA response items. Perhaps unsurprisingly, the optimal window varied across EMA items (with a general minimum of two hours) based on which sensor features were included. This finding highlights the limitations of one-size-fits-all analytic approaches when leveraging passive data from wearables to detect momentary affect and reward functioning. Ultimately, we find that there is signal in passive digital sensing data to detect momentary affect and reward in those with depression and highlight key distinguishing participant characteristics influencing model performance.

## Supplementary Material

ooag005_Supplementary_Data

## Data Availability

The datasets generated and analyzed during the current study are available from the corresponding author upon reasonable request.

## Code availability

The code that supports the findings of this study is available online at https://github.com/akre96/OPTIMA_momentary_ema_detection
