# Supplementary material for: Detecting momentary reward and affect with real-time passive digital sensor data
Source: JAMIA Open. 2026 Jan 23;9(1):ooag005. doi: 10.1093/jamiaopen/ooag005 (PMC12884851; doi:10.1093/jamiaopen/ooag005)
Supplement: ooag005_Supplementary_Data [file ooag005_supplementary_data.docx]

# Supplementary Materials

## Distribution of Ecological Momentary Assessment (EMA) Responses

### **Contextualizing the Population**

The OPTIMA study recruited a population of individuals with moderate or high depression and high, moderate or low levels of anhedonia. To understand the psychometrics of the PVSS in the general population (especially the distribution of anhedonia across the spectrum depression, age, and sex-at-birth) and help establish the study’s inclusion criteria and recruitment strategy, a dataset comprising self-reported PHQ, PVSS, and EMA responses was collected in the planning phase of OPTIMA via Amazon’s Mechanical Turk (MTurk) service (Cohen, Forbes, Khazanov, & Fried, in prep; see https://osf.io/6xnv2/). Amazon MTurk provides access to a diverse online population for survey research, offering a broader demographic sample than our clinical cohort. Target recruitment was n=500, stratified to be equally distributed across five age buckets (18 to 25, 26 to 35, 36 to 45, 46 to 55, 56 to 65) and sex-at-birth (50% male, 50% female). Of 520 responses, 512 have PHQ-8 and PHQ-14 responses, 520 have PVSS responses, and 510 have EMA responses. The distributions of age, sex at birth, PVSS score, PHQ-8 total score (comparable to the PHQ-8 score calculated from the PHQ-14), EMA affect, and reward item responses were compared between this more general sample and the OPTIMA cohort. As the MTurk collected dataset has only one EMA session response, when comparing distributions, the median EMA response is taken per participant per EMA item from the OPTIMA dataset, and the PVSS and PHQ-14 scores are taken from the end of the study.

A Wilcoxon rank-sum test was applied to compare distributions for affect-related EMA items, age, PVSS total score, and PHQ-8 total score. Chi-square independence tests are used to compare distributions for the binary responses to reward-related EMA items and sex at birth. P-values are corrected via Bonferroni adjustment with a familywise error rate (FWER) <0.05. Contextualization results available in supplementary information **Figure S1** and **Table S1**.

Participants in the OPTIMA study were significantly different from the population sampled using MTurk in terms of sex at birth (chi-squared 57.24; Bonferonni adjusted p-value = 1.5e-13), age (mean difference = 3.49 years, Bonferonni adjusted p-value = 1.12e-06), PHQ-8 total score (mean difference 5.68, Bonferonni adjusted p-value = 6.11e-30), and PVSS total score (mean difference = 0.89, Bonferonni adjusted p-value = 2.12e-16). This finding demonstrated that, compared with the population in the MTurk dataset, the OPTIMA study population included participants who were significantly younger, more depressed, more anhedonic, and with more females **(Fig. S1 A**).

Additionally, we see significant differences in the distributions for responses to 7 of 9 affect EMA items and 4 of 6 reward EMA items. These differences are shown in **Figures S1 B-C** and **Table S1.**

**Table S1** EMA response means in the OPTIMA study versus the population sample from Amazon MTurk. P-values from Wilcoxon rank-sum test between OPTIMA and MTurk means adjusted with Bonferroni method FWER < 0.05. * added if adjusted p-value < 0.05

| **EMA item** | **OPTIMA mean** | **MTurk mean** | **Mean difference** | **Adjusted p-value** |
| --- | --- | --- | --- | --- |
| Reward (1-5) | |  |  |  |
| sad | 1.784 | 1.837 | -0.054 | 1.00E+00 |
| stressed*** | 2.427 | 2.078 | 0.348 | 2.01E-08 |
| anxious*** | 2.335 | 1.951 | 0.384 | 6.09E-10 |
| annoyed** | 1.827 | 1.731 | 0.095 | 4.42E-03 |
| energetic*** | 2.114 | 2.839 | -0.725 | 3.57E-18 |
| happy*** | 2.127 | 3.010 | -0.883 | 1.00E-23 |
| motivated*** | 2.198 | 3.310 | -1.112 | 7.28E-40 |
| engaged*** | 2.382 | 3.575 | -1.193 | 2.39E-44 |
| Lonely | 1.776 | 2.010 | -0.234 | 1.00E+00 |
| Affect (0-1) |  |  |  |  |
| anticipatory*** | 0.480 | 0.735 | -0.255 | 4.11E-11 |
| consummatory*** | 0.427 | 0.715 | -0.289 | 2.07E-14 |
| effort*** | 0.302 | 0.639 | -0.336 | 8.55E-17 |
| inactive enjoy | 0.169 | 0.265 | -0.096 | 6.89E-02 |
| inactive effort | 0.253 | 0.346 | -0.093 | 1.55E-01 |
| meaning*** | 0.359 | 0.715 | -0.356 | 2.25E-19 |

**Figure S1** Distribution of demographics, depression severity, anhedonia, and EMA responses between OPTIMA and a population sample collected on Amazon MTurk. **A**) Sex at birth, age, PHQ-8 score (depression severity, directly comparable to PHQ-14 total score), PVSS (anhedonia; lower means more anhedonia). All measures significantly difference at p < 0.001 between groups. **B)** Bar plot comparing affect EMA item response and **C**) reward EMA responses. Significant differences between items in **B** and **C** annotated if p < 0.05 after Bonferroni adjustment with FWER < 0.05. Annotation legend: * < 0.05, ** < 0.01, *** < 0.001.

**
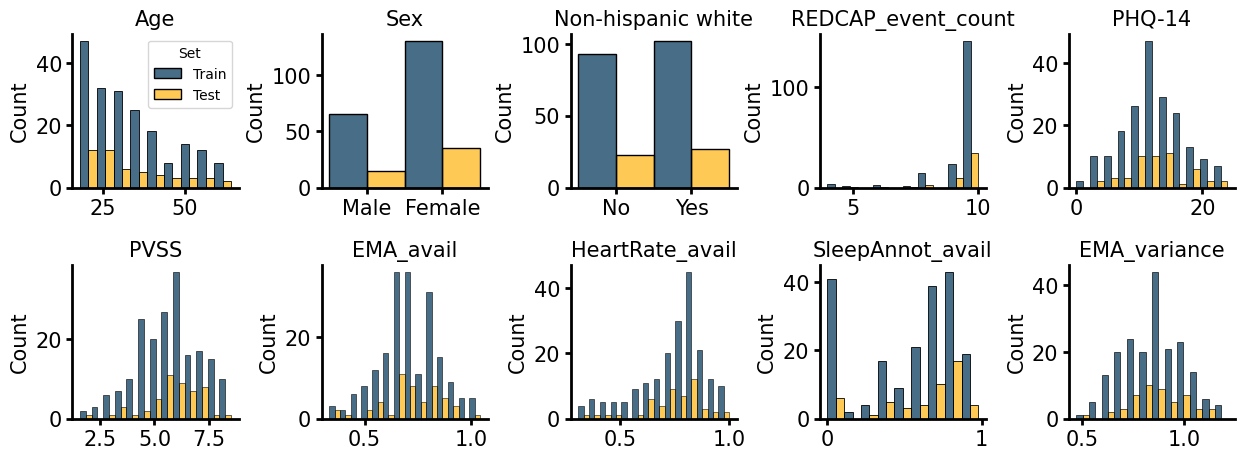
**

**Figure S2** Histograms of train and test set user characteristics.

Sensitivity to Missing Data

To assess the sensitivity of the models to missing data, the test set performance was evaluated on the full test set as well as a subset of EMA sessions missing fewer than 10 of 29 features (n=2716, 56.6% of EMA sessions in the test set). Differences between the AUROC on the full test set relative to the high data availability test set are compared per outcome via a Wilcoxon rank-sum test corrected for multiple testing via the Bonferroni adjustment with the FWER set to 0.05.

Results indicate significant differences in performance based on data availability. For the ‘anxious’ and ‘energetic’ EMA items, which perform above random chance, models performed significantly better on the full sample than on the subset with fewer missing features, suggesting that the highly missing features are not essential for these predictions. In contrast, model performance for the ‘consummatory,’ ‘sad,’ ‘inactive enjoy,’ ‘anticipatory,’ and ‘meaning’ EMA items was higher in the subset with less missing data, indicating that data completeness may enhance prediction accuracy for these outcomes. The full results of the sensitivity analysis are summarized in **Table S2**. The findings may be explained in part by the fact that models whose performance degrades, such as the model detecting sad EMA, rely on features that are sampled less frequently, such as HRV (see **Figure S3**).

**
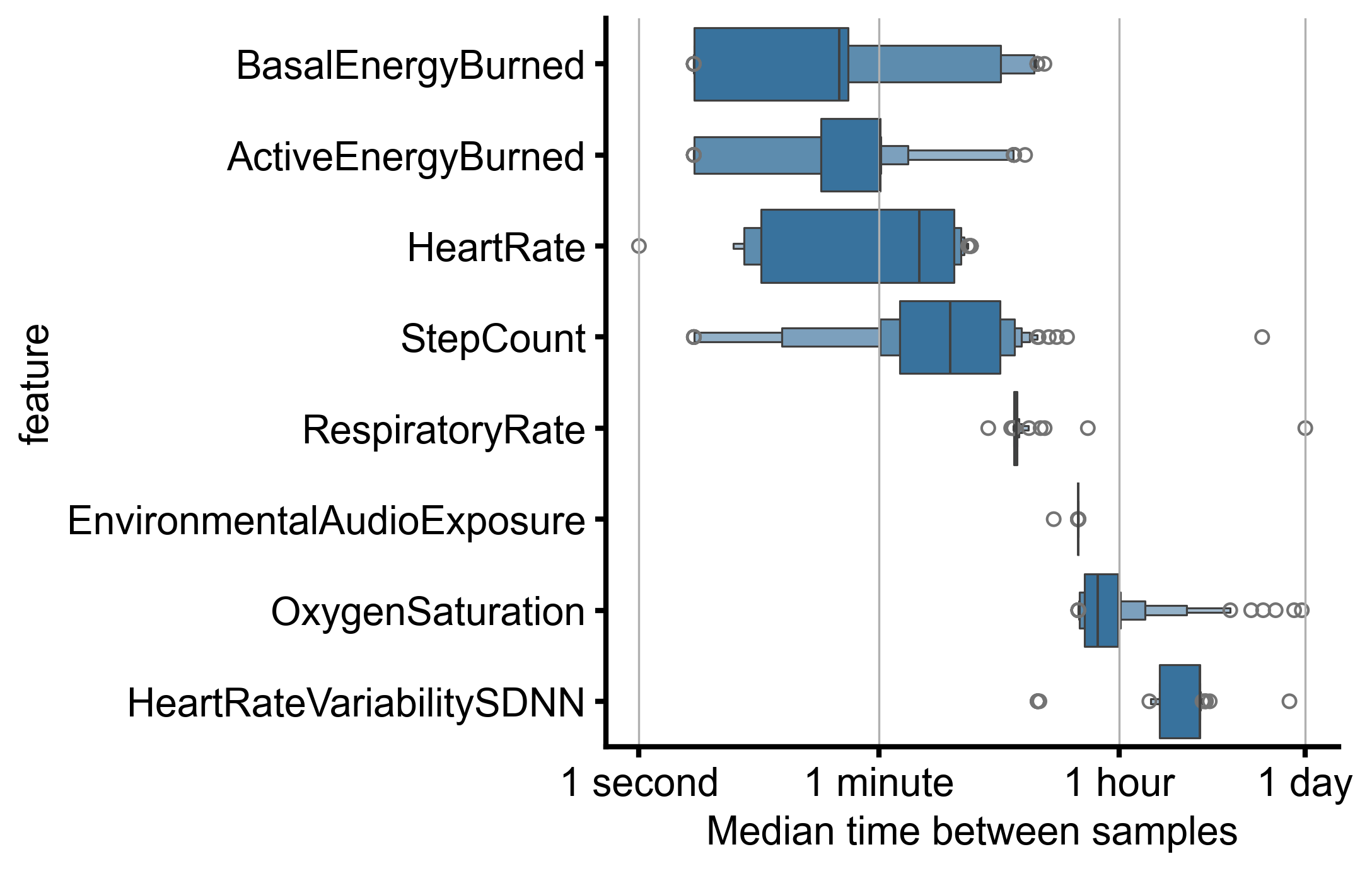
**

**Figure S3** Median time between samples of a feature per user. Boxen plot shows distribution across participants. X-axis is logarithmically scaled.

**
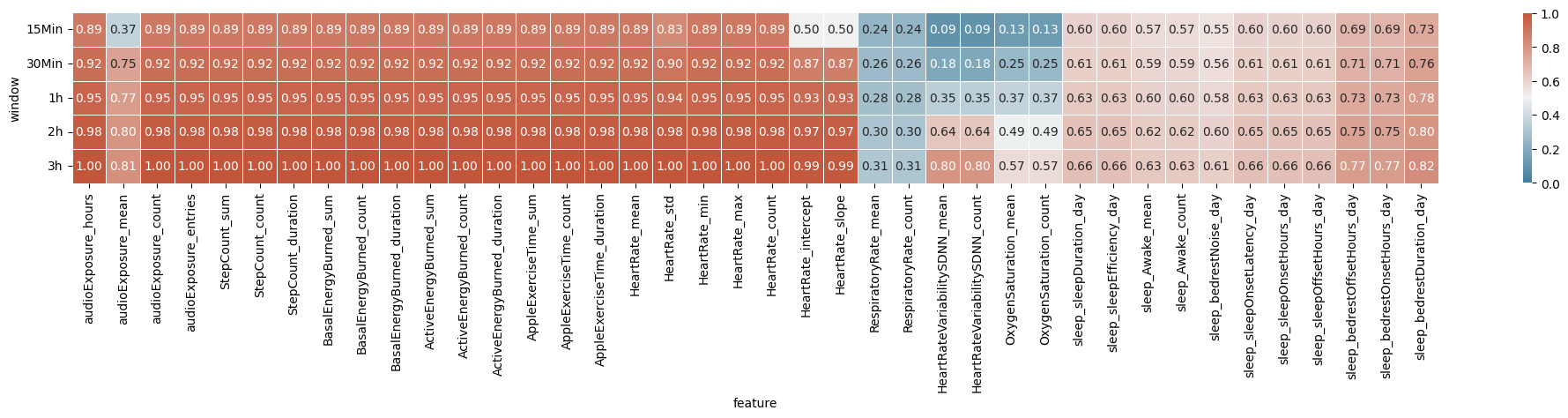
**

**Figure S4** Missing passive sensor features based on availability of data prior to EMA session start. Note Sleep features refer to availability of the prior night’s sleep data. A value of 1 indicates all data is available (0% missing) and a value of 0 indivates no data is available (100% missing).

**Table S2** Difference in performance of models detecting EMA item response in the test set on either the full test set, or a subset of responses with fewer than 10 of 29 features missing. Bold values indicate FWER adjusted p-value < 0.05 for difference and the full sample model performed greater than random chance (AUROC > 0.5 and adjusted p-value < 0.05).

| **EMA Item** | **Features** | **Window** | **Mean AUROC** | | **AUROC Difference** | **Adjusted P-value** |
| --- | --- | --- | --- | --- | --- | --- |
|  |  |  | **Full Test Set** | **Missing < 10 features** |  |  |
| anxious | momentary | 2h | 0.545 | 0.510 | 0.035 | **1.59E-26** |
| consummatory | with sleep | 2h | 0.543 | 0.559 | -0.016 | **9.04E-17** |
| sad | momentary | 3h | 0.572 | 0.584 | -0.012 | **1.18E-11** |
| inactive enjoy | with sleep | 2h | 0.551 | 0.563 | -0.012 | **5.13E-10** |
| anticipatory | momentary | 2h | 0.549 | 0.557 | -0.008 | **1.68E-04** |
| meaning | with sleep | 2h | 0.532 | 0.539 | -0.007 | **2.88E-04** |
| energetic | with sleep | 3h | 0.586 | 0.580 | 0.006 | **1.54E-02** |
| annoyed | momentary | 2h | 0.540 | 0.536 | 0.005 | 5.13E-02 |
| motivated | with sleep | 3h | 0.567 | 0.562 | 0.005 | 1.53E-01 |
| stressed | with sleep | 1h | 0.554 | 0.556 | -0.002 | 1.00E+00 |
| inactive effort | with sleep | 2h | 0.550 | 0.551 | -0.001 | 1.00E+00 |

## Training Set Best Models

Performance of models in the training set are shown in **Figure S5.** To compare those models with the best performance in the test dataset, those models training performance is highlighted in **Table S3.** The high AUROC on training for detecting the “happy” EMA suggests this model may have overfit resulting in the observed poor test set performance.


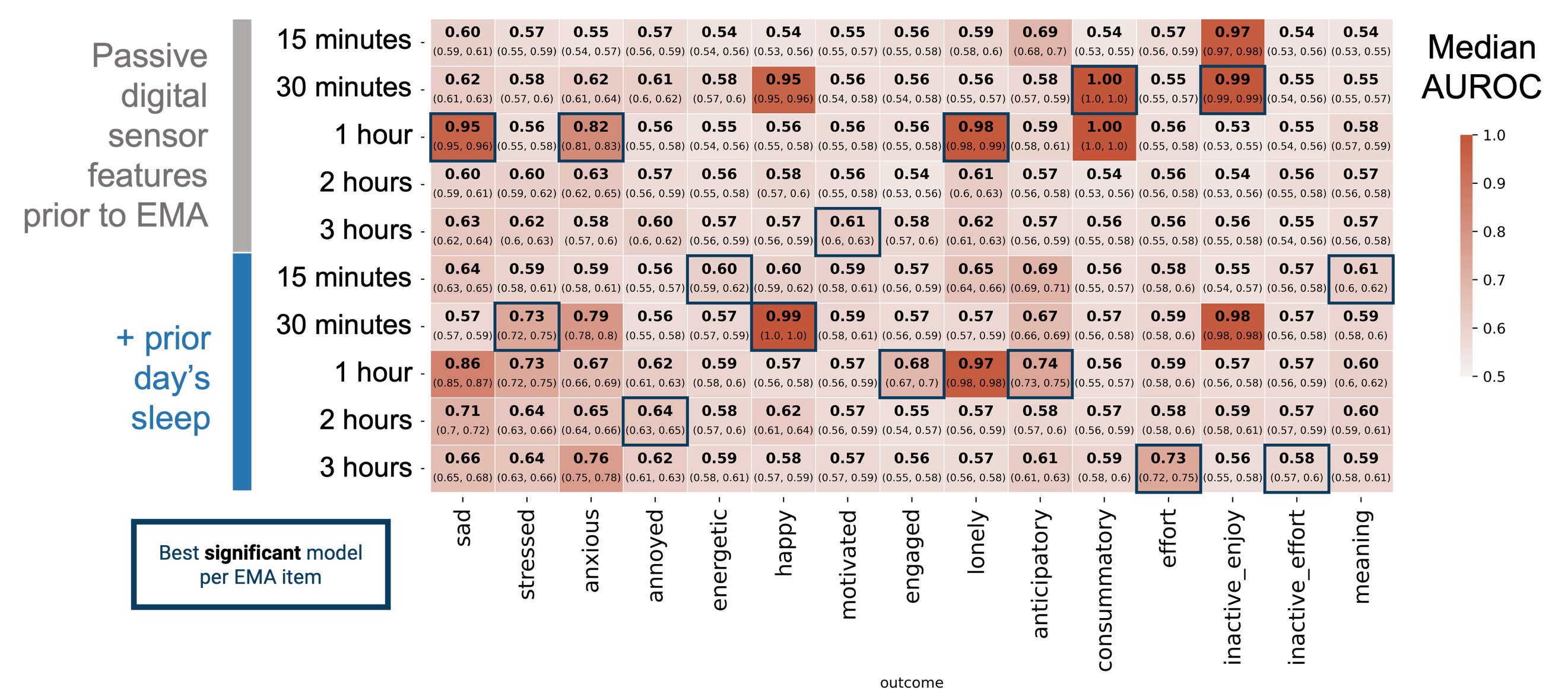


**Figure S5** Model performance using passive digital sensor data to detect EMA item response (median AUROC ± 95% Bonferroni-adjusted bootstrapped confidence interval) on the training set of users for each feature set (rows) and each outcome (columns). Y-axis separates different feature sets used as inputs to the models based on aggregation window (15 minutes to 3 hours) and the inclusion of passively sensed sleep from the night before. Bold values indicate model AUROC performance for an outcome where p<0.05 for testing that AUROC > 0.5 after Bonferroni adjustment of Mann Whitney U-test. Blue outline indicates highest median AUROC for significantly performing model for a given outcome column in the training set. Note that apparent equal values only appear as such due to rounding to two decimal places. AUROC = Area under the receiver operator characteristic curve.

**Table S3.** Training set performance for best models at detecting each EMA item using passive digital sensing data in the test set. Performance reported as median AUROC value across 1000 bootstraps in the training set. Train N refers to number of available EMA responses with associated passive digital sensor data in the training set. AUROC = Area under the receiver operator characteristic curve.

| **EMA Item** | **Feature Set** | **Model** | **AUROC (95% CI)** | **Train N** |
| --- | --- | --- | --- | --- |
| sad | Momentary 3h | XGBoost | 0.633 (0.619, 0.645) | 17707 |
| stressed | With Sleep 1h | LightGBM | 0.736 (0.719, 0.750) | 17712 |
| anxious | Momentary 2h | Random Forest | 0.633 (0.621, 0.648) | 17719 |
| annoyed | Momentary 2h | LightGBM | 0.573 (0.561, 0.586) | 17715 |
| energetic | With Sleep 3h | Random Forest | 0.593 (0.580, 0.607) | 17713 |
| happy | Momentary 30Min | LightGBM | 0.957 (0.951, 0.961) | 17719 |
| motivated | With Sleep 3h | Random Forest | 0.579 (0.566, 0.594) | 17718 |
| engaged | With Sleep 2h | XGBoost | 0.557 (0.545, 0.574) | 17717 |
| lonely | Momentary 2h | LightGBM | 0.614 (0.602, 0.626) | 17719 |
| anticipatory | Momentary 2h | Random Forest | 0.570 (0.557, 0.582) | 17647 |
| consummatory | With Sleep 2h | Random Forest | 0.572 (0.560, 0.585) | 17640 |
| effort | With Sleep 3h | LightGBM | 0.736 (0.722, 0.747) | 17641 |
| inactive_enjoy | Momentary 30Min | LightGBM | 0.992 (0.991, 0.994) | 17640 |
| inactive_effort | With Sleep 3h | Random Forest | 0.584 (0.572, 0.597) | 17633 |
| meaning | Momentary 3h | XGBoost | 0.537 (0.510, 0.562) | 17640 |

**Table S4.** Extreme response performance for best models at detecting each EMA item using passive digital sensing data in the test set for those where likert 1 to 5 responses were binarized. Extreme response is defined as a response of 1 or 5 on the likert scale. Performance reported as median AUROC value across 100 bootstraps in the test set. Train N refers to number of available EMA responses with associated passive digital sensor data in the training set. AUROC = Area under the receiver operator characteristic curve.

| **EMA Item** | **Feature Set** | **Model** | **AUROC (95% CI)** | **Test N** | **Extreme N** |
| --- | --- | --- | --- | --- | --- |
| sad | Momentary 3h | XGBoost | 0.710 (0.672, 0.746) | 1879 | 130 |
| stressed | With Sleep 1h | LightGBM | 0.584 (0.549, 0.614) | 1104 | 195 |
| anxious | Momentary 2h | Random Forest | 0.567 (0.540, 0.596) | 1084 | 117 |
| annoyed | Momentary 2h | LightGBM | 0.566 (0.520, 0.616) | 1953 | 107 |
| energetic | With Sleep 3h | Random Forest | 0.633 (0.590, 0.688) | 1392 | 26 |
| happy | Momentary 30Min | LightGBM | 0.543 (0.494, 0.583) | 1210 | 61 |
| motivated | With Sleep 3h | Random Forest | 0.498 (0.458, 0.550) | 1246 | 42 |
| engaged | With Sleep 2h | XGBoost | 0.520 (0.493, 0.552) | 941 | 70 |

## Basal Energy Expenditure

To investigate what basal energy expenditure may represent, **Figure S5** illustrates the median value of basal energy expenditure across a 24-hour period split by users’ sex at birth. The observed sex-based differences in basal energy expenditure suggest that models may capture underlying demographic differences, affecting prediction accuracy for each group.


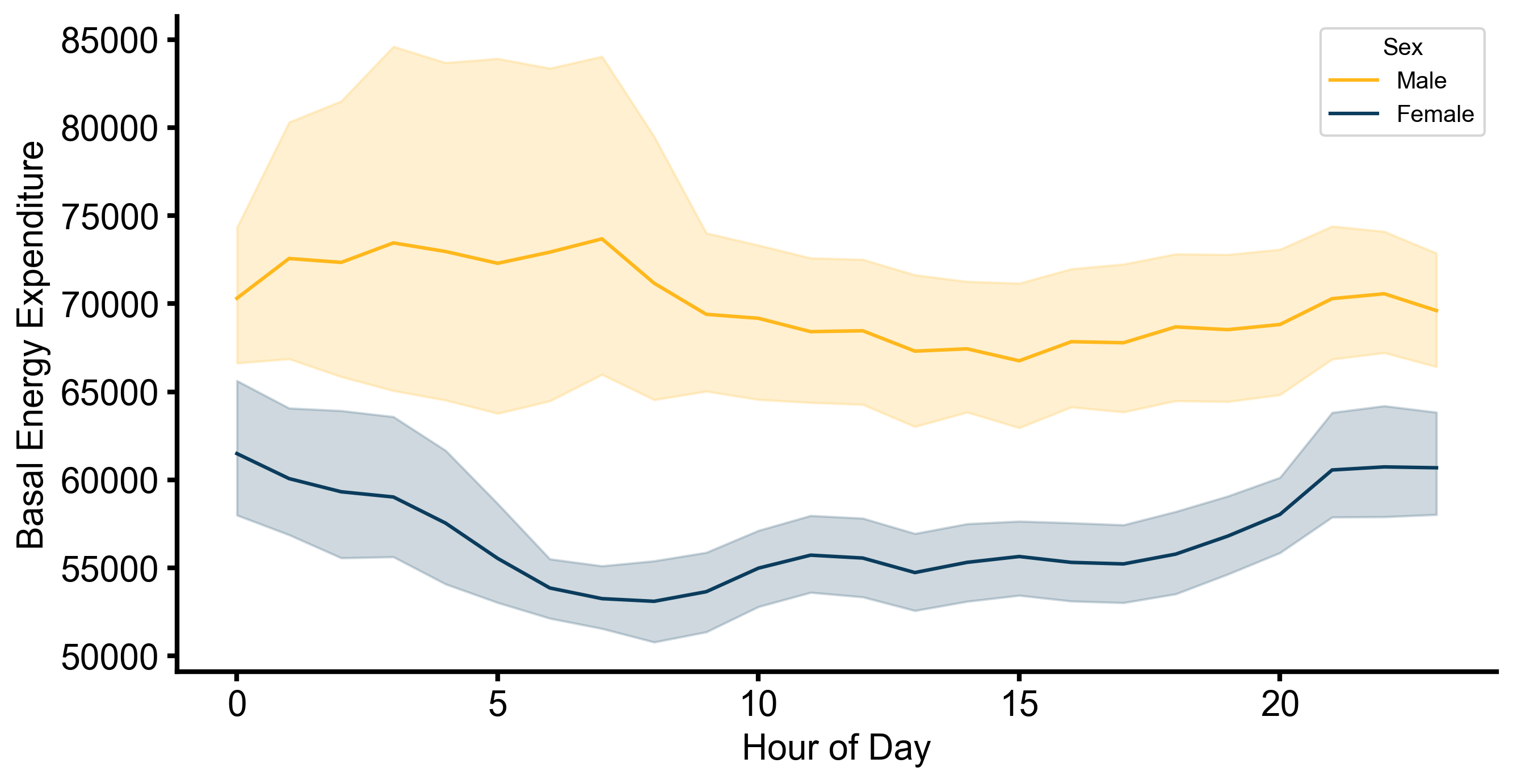


**Figure 5** Median value per user of basal energy expenditure per hour of the day split by sex at birth. Error bands represent 95% confidence intervals.
